# Accelerating metadata annotation in collaborative research centers: a hybrid AI workflow for biomedical entities

**DOI:** 10.1093/bioadv/vbag283

**Published:** 2026-09-23

**Authors:** Manuel Watter, Felix Engel, Aref Kalantari, Claudia Giuliani, Karin Schuller, Claus-Werner Franzke, Markus Sperandio, Harald Binder, Klaus Kaier

**Affiliations:** Institute of Medical Biometry and Statistics, Medical Faculty and Medical Center, University of Freiburg, Freiburg, Germany; Institute of Medical Biometry and Statistics, Medical Faculty and Medical Center, University of Freiburg, Freiburg, Germany; Institute of Medical Biometry and Statistics, Medical Faculty and Medical Center, University of Freiburg, Freiburg, Germany; Institute of Medical Biometry and Statistics, Medical Faculty and Medical Center, University of Freiburg, Freiburg, Germany; Research Data Management Scientific IT, Helmholtz München, München, Germany; Institute for Infection Prevention and Control, Medical Faculty and Medical Center, University of Freiburg, Freiburg, Germany; Institute of Cardiovascular Physiology and Pathophysiology, Biomedical Center, Ludwig-Maximilians-Universität München, München, Germany; Institute of Medical Biometry and Statistics, Medical Faculty and Medical Center, University of Freiburg, Freiburg, Germany; Center for Integrative Biological Signaling Studies (CIBSS), University of Freiburg, Freiburg, Germany; Institute of Medical Biometry and Statistics, Medical Faculty and Medical Center, University of Freiburg, Freiburg, Germany

## Abstract

**Background:**

Collaborative Research Centers rely on FAIR-compliant, richly structured metadata, yet manual annotation is a major bottleneck. We implemented a search-augmented large language model (LLM) workflow within a local research data management system to pre-annotate biomedical entities, using human-in-the-loop verification to ensure data quality.

**Methods:**

The pipeline uses Gemini 3 Pro in a two-step prompting strategy: (1) identify dataset deposits and stable identifiers in articles converted to Markdown; (2) extract structured fields from curated repository landing pages rendered via a headless browser. To handle a highly hierarchical metadata schema, we flattened the schema for prompting and remapped outputs to strict JSON with granular provenance tags. Authors received pre-filled metadata and could accept, edit, or delete entries (TP, FP, FN mapping). Performance metrics (precision, recall, F1) were estimated as proportions and synthesized via random-effects meta-analysis.

**Results:**

Among 51 screened articles, the LLM identified a repository deposit in 31; authors responded for 17 (55% of articles with an identified deposit; 33% of all screened articles), yielding 39 verified datasets. False positives were rare (mean 0.23) and false negatives low (mean 1.46). Precision was consistently high at 99.65% (95% CI 98.42%–100.00%). Recall showed more variability at 93.75% (95% CI 89.79%–96.96%), and the F1 score reached 96.17% (95% CI 93.78%–98.11%).

**Conclusions:**

The hybrid workflow achieved high author-verified precision with moderate recall, shifting effort from drafting to reviewing while maintaining schema compliance. As these metrics rely on author acceptance and response rates were modest, broader, multi-site validation is needed.

## 1 Introduction

Long-term research hubs like Germany’s Collaborative Research Centers (CRCs) rely on structured data sharing for FAIR-compliant reuse (Wilkinson *et al.* 2016, Kanza and Knight 2022). This necessity has intensified due to the massive growth of digital information, often termed a ‘data deluge’, which has shifted research data management from an optional task to a core scientific requirement (Engel *et al.* 2026). In biomedicine, tagging entities such as organisms or genes boosts discoverability. Since manual annotation requires considerable effort, automated named entity recognition is often used to classify terms (for instance, identifying “mouse” as an “organism”) (Nadeau and Sekine 2009). Because each CRC has distinct scientific requirements, a dedicated metadata schema must first be developed to reflect its specific experimental context (Engel *et al.* 2026). The specific targets depend on the experimental focus. Cell lines matter for in-vitro assays, but they’re irrelevant in population studies. Only once this schema is in place can researchers begin manually annotating their datasets. This ‘metadata bottleneck’ frequently leads to the reality of ‘empty archives’, as researchers often prioritize immediate research demands over labor-intensive documentation (Watter *et al.* 2023, Engel *et al.* 2026). While research data management is indispensable, it should be designed to be as effortless and unobtrusive as possible, enabling researchers to concentrate on their scientific work.

We outline the implementation of the AI-augmented workflow, into an existing research data management system (Watter *et al.* 2023). The AI system is an LLM-based, search-augmented pipeline designed to identify and annotate biomedical entities, particularly focusing on handling multiple datasets across different public repositories. This workflow uses a two-step strategy that first identifies datasets, then extracts data primarily from curated repository landing pages as the source of truth.

The target environment, fredato, is a standardized metadata collection system built as a wrapper over GitLab and Nextcloud (Watter *et al.* 2023), utilized by Collaborative Research Centers. This existing system already relies on a specialized, hierarchical metadata schema defined using a bottom-up approach with domain experts (Engel *et al.* 2025, Kaier *et al.* 2026). This strategy balances the need for consortium-wide standardization with the high granularity required for specialized biomedical data, such as disease models and readouts (Watter *et al.* 2023, Engel *et al.* 2026).

We aim to implement a hybrid model (Anthuvan *et al.* 2025), where LLMs accelerate the data documentation process (Watter *et al.* 2023, Reddy 2024, Anthuvan *et al.* 2025). By deploying the model as a pre-annotation assistant, the system overcomes the ‘blank slate problem’ and significantly reduces ‘creation fatigue’ for professional researchers (Wang *et al.* 2024). To be effective, the AI workflow must adhere to the existing schema’s logic (Kaier *et al.* 2026), while maintaining data quality through human validation (Reddy 2024, Anthuvan *et al.* 2025).

We emphasise that the contribution reported here is a workflow rather than a model. The pipeline uses an off-the-shelf foundation model without training, fine-tuning or adapter layers; what is engineered is the orchestration around it–a two-step prompting strategy that separates dataset identification from field extraction, retrieval of curated repository landing pages as the designated source of truth, flattening of a hierarchical schema for prompting with strict remapping to validated JSON, granular provenance tagging of every metadata object, and a technically enforced human-in-the-loop validation step. Figure 1 shows this architecture. The evaluation is framed accordingly: it asks how well the orchestration performs in production, not how well a particular model performs in isolation.

**Figure 1:**
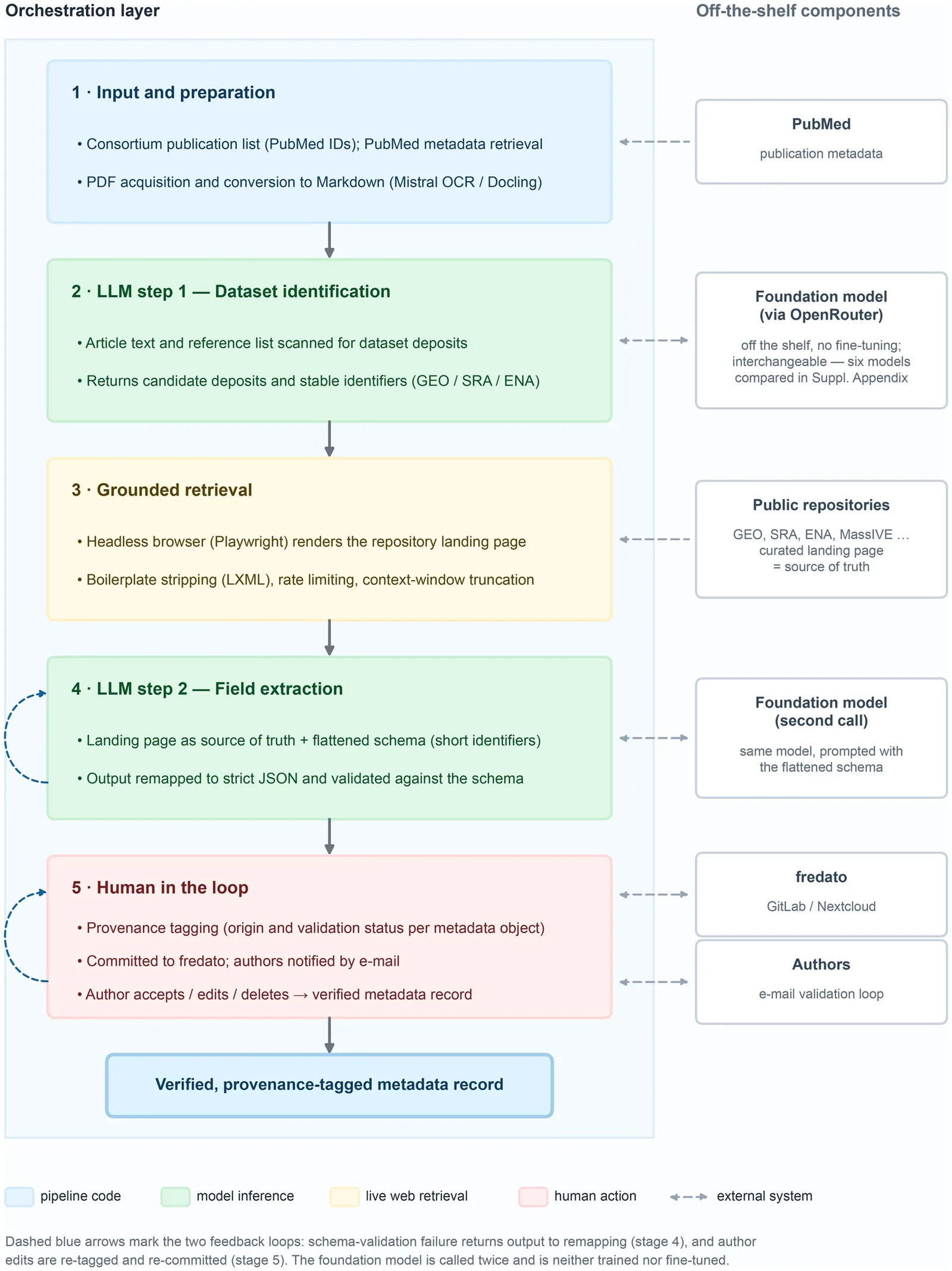
Architecture of the AI-augmented metadata annotation workflow.

## 2 Methods

The implementation of an AI-augmented metadata annotation workflow is based on the search-augmented LLM pipeline that was tested previously (Kaier *et al.* 2026), and the existing metadata annotation framework within the fredato research data management system (Watter *et al.* 2023). The study follows a human-in-the-loop approach (Supplementary Appendix 1 for the detailed operational workflow), where artificial intelligence functions as a pre-annotation assistant to accelerate traditionally labor-intensive documentation processes while remaining under the epistemic oversight of the responsible scientist (Wang *et al.* 2024, Anthuvan *et al.* 2025).

The pipeline uses Gemini 3 Pro to identify and annotate biomedical entities from public repositories via a two-step prompting strategy. All model calls were routed through the OpenRouter API under the provider model identifier google/gemini-3-pro-preview, the identifier under which this model was served on 18 November 2025; the provider exposed no finer snapshot or release tag in the response payload, and the identifier has since been retired, so the exact snapshot can no longer be addressed. Sampling parameters were left at the documented provider defaults; temperature, top_p and seed were not pinned. This reflects realistic deployment conditions and acknowledges that pinning these parameters would not render the pipeline deterministic. Providers may silently route requests to quantised or substituted model variants under load; repository landing pages are rendered at request time and can carry per-request content (NCBI GEO pages, for example, embed a session identifier that enters the prompt together with the remaining page text); and normalisation behaviour can depend on the composition of the inference batch in which a request is processed. Rather than reporting a nominal configuration that cannot be enforced end to end, we characterise run-to-run variability empirically. To enable accurate text processing, PDF manuscripts were first converted into Markdown format (using Mistral OCR or Docling). First, the model scans the formatted text and references to identify dataset deposits and capture stable identifiers such as GEO or SRA URLs. In the second step, the model visits the curated repository landing pages, treated as the primary source of truth. Retrieval was performed using a headless browser (Playwright) to render dynamic JavaScript content. The extracted HTML was programmatically cleaned to remove boilerplate elements (e.g. navigation bars, styling) and truncated to fit within the model’s context window (detailed cleaning specifications and fallback mechanisms are described in Supplementary Appendix 1, available at supplementary material  *Bioinformatics Advances* online) to populate fields in the specialized, hierarchical metadata schema.

The AI-generated suggestions are then integrated into fredato, a web-based metadata editor built as a wrapper over GitLab and Nextcloud that utilizes JSON schema definitions. To ensure data integrity and distinguish between human and machine inputs, the system implements a granular provenance tracking mechanism that tags individual metadata objects with their origin and validation status (see Supplementary Appendix 1, available at supplementary material  *Bioinformatics Advances* online): To prevent context poisoning caused by the schema’s complexity, we employed a schema flattening strategy. Instead of feeding the full JSON structure to the LLM, the schema was converted into a tree-like textual representation using short identifiers. The model’s output was then mapped back to the strict JSON format. This implementation respects the hierarchical and conditional logic of the domain-specific metadata schema (Engel *et al.* 2025), where specific entries (e.g. selecting a particular “tissue source”) trigger the requirement for refined sub-fields. Within the interface, authors are presented with pre-filled metadata fields. In an email-based interactive validation loop, the model’s outputs are presented to the authors of the articles as editable suggestions rather than final records. Authors act as the “human in the loop,” reviewing the suggestions with the ability to accept, modify, or delete entries. This step ensures the final data remains accurate, allowing scientists to correct potential model hallucinations or generalizations (Wang *et al.* 2024; Anthuvan *et al.* 2025). The design choice of treating the curated repository landing page as the source of truth was validated previously within the same pipeline family: repository-only extraction was more precise than repository-plus-article extraction across all models tested (91.9% vs. 88.3%, *P* = 0.004; (Kaier *et al.* 2026). Schema flattening and strict JSON remapping are prerequisites rather than optional enhancements, since serialising the full hierarchical schema exceeds the practical context budget and induces context poisoning, and unvalidated output cannot be written into the metadata repository; provenance tagging is applied after extraction and cannot affect extraction accuracy.

The primary objective of the evaluation is to validate the LLM’s performance against high-quality labels generated by subject matter experts (the authors) during the validation phase. By tracking how authors interact with the pre-filled forms, we derive effect measures based on specific user actions. We mapped user actions to performance metrics: accepted suggestions count as True Positives (TP), deleted suggestions as False Positives (FP), manually added entities as False Negatives (FN). These quantities are derived from author interactions with a pre-filled form and therefore measure acceptance and editing behaviour rather than agreement with an annotation produced independently of the model. Because authors form their judgement only after seeing the model output, the design is exposed to anchoring and to a default-acceptance tendency, and a false negative is recorded only where an author proactively adds a missing entity. We therefore refer to these quantities as author-verified precision, recall and F1 throughout, and interpret them as an upper bound on extraction performance rather than as unbiased estimates. To bound the same quantities from below, Supplementary Appendix 2, available at supplementary material  *Bioinformatics Advances* online reports a second, independent evaluation in which the annotations recorded during the schema-development process serve as a fixed reference standard and every deviation from that reference is charged as an error without adjudication.

Based on these interactions, the following performance metrics are calculated:

**Precision:** Proportion of accepted suggestions. $\mathrm{Precision}=\frac{TP}{TP+FP}$

**Recall:** Ability to identify required entities. $\mathrm{Recall}=\frac{TP}{TP+FN}$

**F1 Score:** Harmonic mean of precision and recall. $F1=\frac{2*TP}{2*TP+FN+FP}$

This design provides a structured environment in which the responsible author acts as the reference annotator for every schema field, which constrains the otherwise open-ended nature of recall measurement in scientific annotation (Pangakis *et al.* 2023, Kaier *et al.* 2026). It does not, however, provide an annotation that is independent of the model output, and the resulting recall is therefore an upper bound (see Supplementary Appendix 2, available at supplementary material  *Bioinformatics Advances* online). Because the evaluation is performed over a highly hierarchical metadata schema (342 levels, see (Engel *et al.* 2026, Kaier *et al.* 2026)), most fields are correctly left blank, yielding a very large number of true negatives. Metrics that heavily incorporate true negatives (such as accuracy) are therefore not reported, as they are less informative for the intended decision context.

To evaluate the effectiveness of the AI-augmented workflow, the three performance metrics, Precision, Recall, and F1 Score are analyzed using the statistical methodology used in previous approaches (Giuliani *et al.* 2025, Kaier *et al.* 2026, Watter *et al.* 2026). In detail, these metrics are treated as proportions derived from the comparison between the initial AI-generated suggestions and the final “gold standard” labels validated by the authors within the fredato system. To estimate the overall performance across the different research articles and datasets, meta-analyses of single proportions are conducted (Schwarzer and Rücker 2022). Due to the expected small sample sizes per article and the likelihood of proportions being near 1, the Freeman–Tukey double arcsine transformation is applied to stabilize the variance (Miller 1978). A random-effects model using the restricted maximum likelihood (REML) estimator is employed to calculate aggregate estimates and their corresponding 95% confidence intervals, accounting for potential variability between different scientific domains or metadata tasks. An independent evaluation on a subsample of 10 datasets for which model-independent expert annotations were available from the schema-development process is reported in Supplementary Appendix 2, available at supplementary material  *Bioinformatics Advances* online, using the same effect measures and the same random-effects specification.

Because several datasets can originate from the same article, the 39 datasets are not independent observations. As a sensitivity analysis, we therefore re-estimated all three metrics using a mixed-effects Poisson regression in which the numerator of the respective metric is the outcome, the corresponding denominator enters as exposure, a random intercept accounts for the article, and standard errors are clustered by article.

## 3 Results

The workflow was rolled out in December 2025, with reminder emails sent at 5 weeks and 10 weeks after rollout. Figure 2 summarizes the yield of the multi-step workflow across 51 screened articles (40 original articles and 11 review articles). A repository deposit was identified by the LLM for 31 articles; for 17 of these articles, authors provided feedback (55% of the 31 articles with an identified deposit, corresponding to 33% of all 51 screened articles), enabling human-in-the-loop verification of the LLM suggestions for 39 datasets. For the remaining pathway, 9 articles had no repository identified by the LLM; in this branch, only one author provided additional information for 1 unpublished dataset; however, from four articles in the repository pathway, 11 unpublished datasets were added on top of the datasets already published in public repositories. No human verification was obtained at the time of writing for a total of 22 articles (Fig. 2).

**Figure 2:**
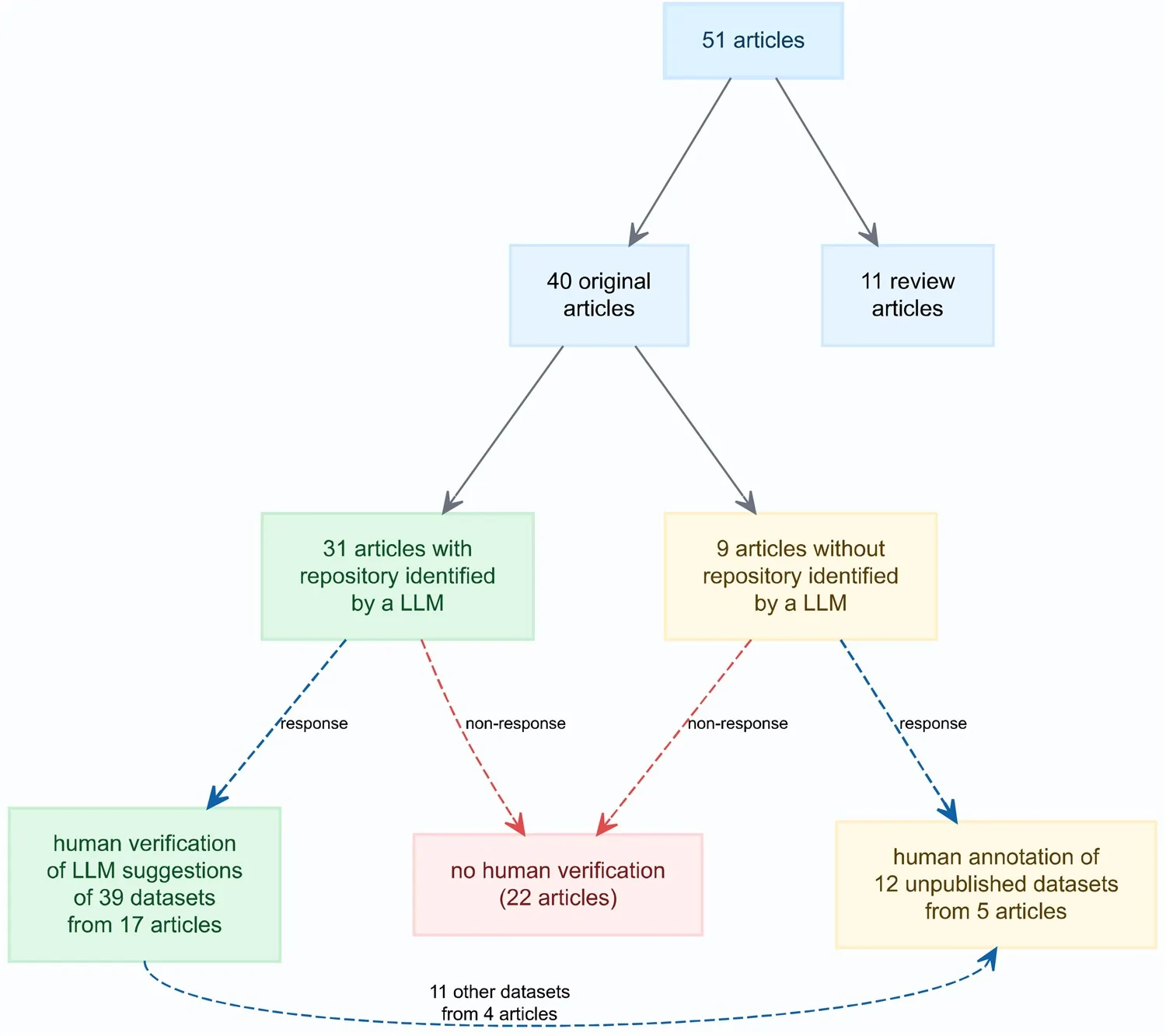
Yield of the multi-step LLM workflow across 51 screened articles.

Performance evaluation was based on the 39 datasets with human verification. Across these datasets, the number of true positives (TP) averaged 13.15 (*SD* 4.57; range 6–27). False positives (FP) were rare, with a mean of 0.23 (*SD* 0.58; range 0–2). False negatives (FN) were also low, with a mean of 1.46 (*SD* 1.93; range 0–6). In absolute terms, the 39 datasets yielded 513 true positives, 9 false positives and 57 false negatives.

Author-verified precision was consistently high across datasets (mostly 100%), with an overall random-effects estimate of 99.65% (95% CI 98.42%–100.00%) and no detectable heterogeneity (*I*² = 0.00%; Fig. 3).

**Figure 3:**
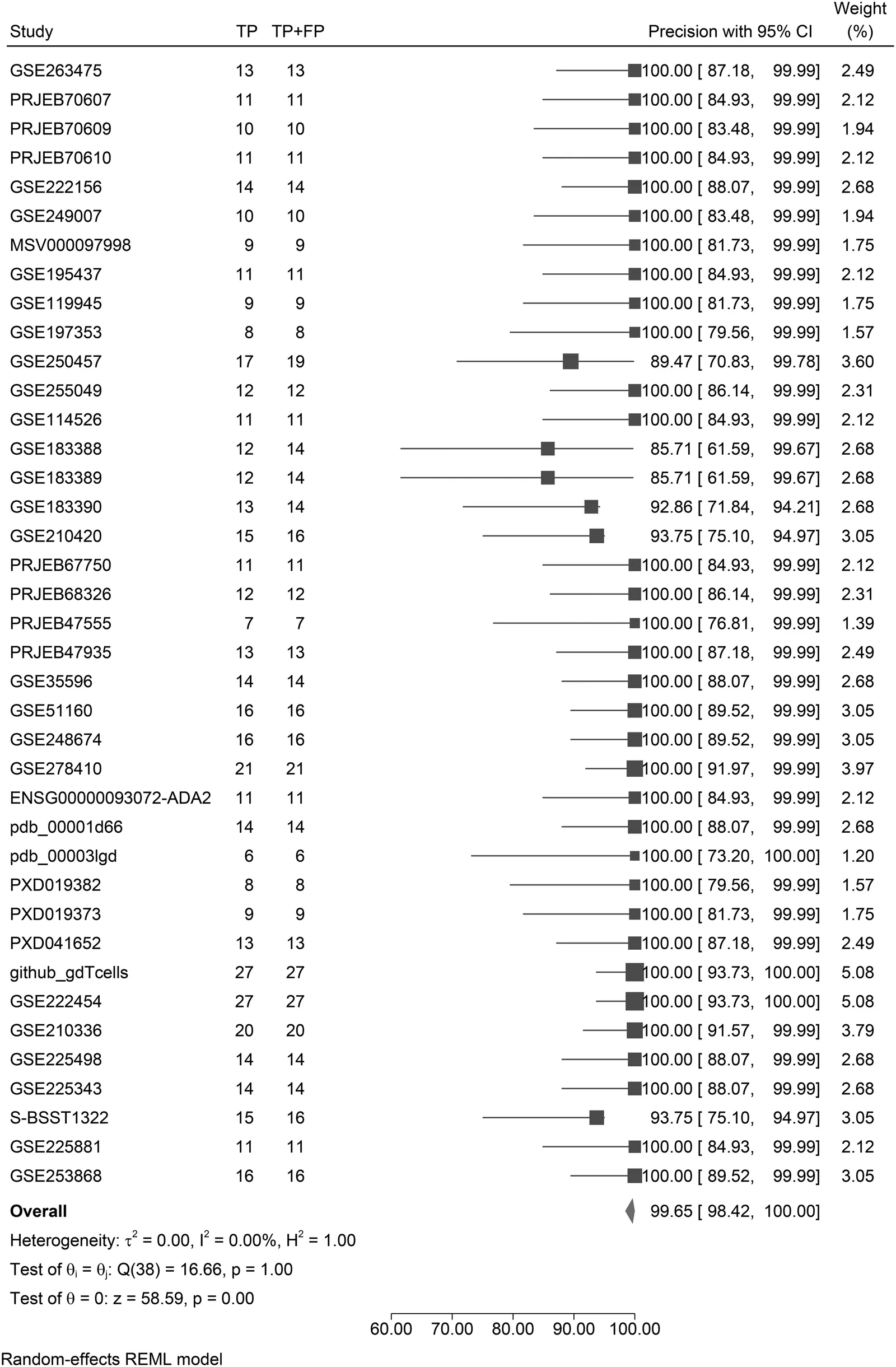
Precision of the multi-step LLM workflow according to the human-in-the-loop verification of LLM suggestions.

Recall showed more variability, with an overall estimate of 93.75% (95% CI 89.79%–96.96%) and moderate heterogeneity (I² = 55.08%; Fig. 4). The combined performance, expressed as the F1 score, yielded an overall estimate of 96.17% (95% CI 93.78%–98.11%) with moderate heterogeneity (*I*² = 61.16%; Fig. 5). Accounting for the clustering of datasets within articles did not change these conclusions. In mixed-effects Poisson models with a random intercept for article and standard errors clustered by article (39 datasets in 17 articles), the estimated precision was 98.28% (95% CI 96.13%–100.47%; the Poisson specification does not constrain the fitted proportion to 100%), recall 90.00% (95% CI 83.60%–96.90%) and F1 93.96% (95% CI 90.36%–97.69%). The estimated between-article variance was negligible in all three models, indicating no detectable within-article correlation beyond that already accommodated by the random-effects meta-analysis. The mixed-model estimates are marginally lower than the meta-analytic estimates because they weight datasets by their denominator rather than approximately equally.

**Figure 4:**
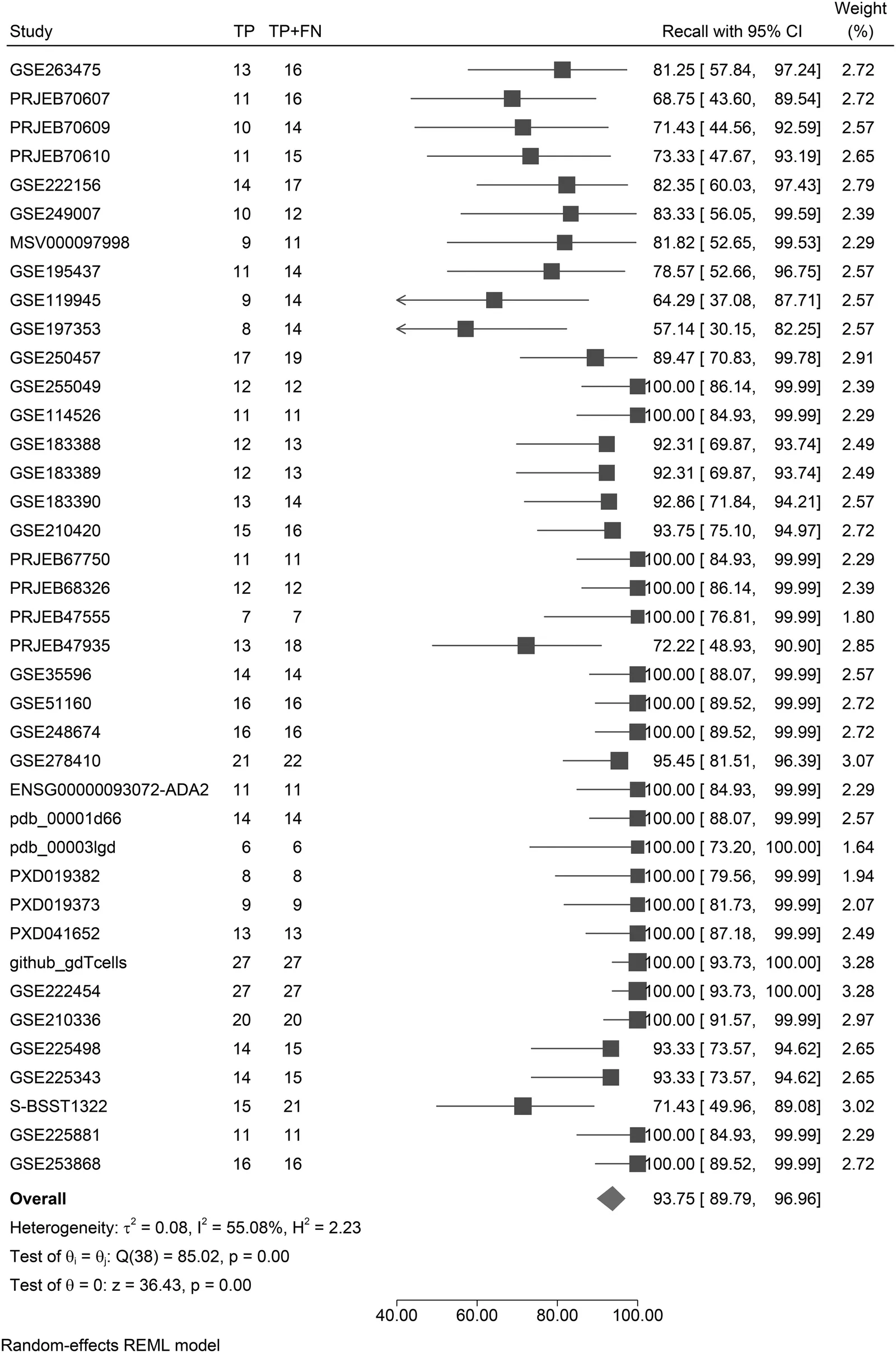
Recall of the multi-step LLM workflow according to the human-in-the-loop verification of LLM suggestions.

**Figure 5:**
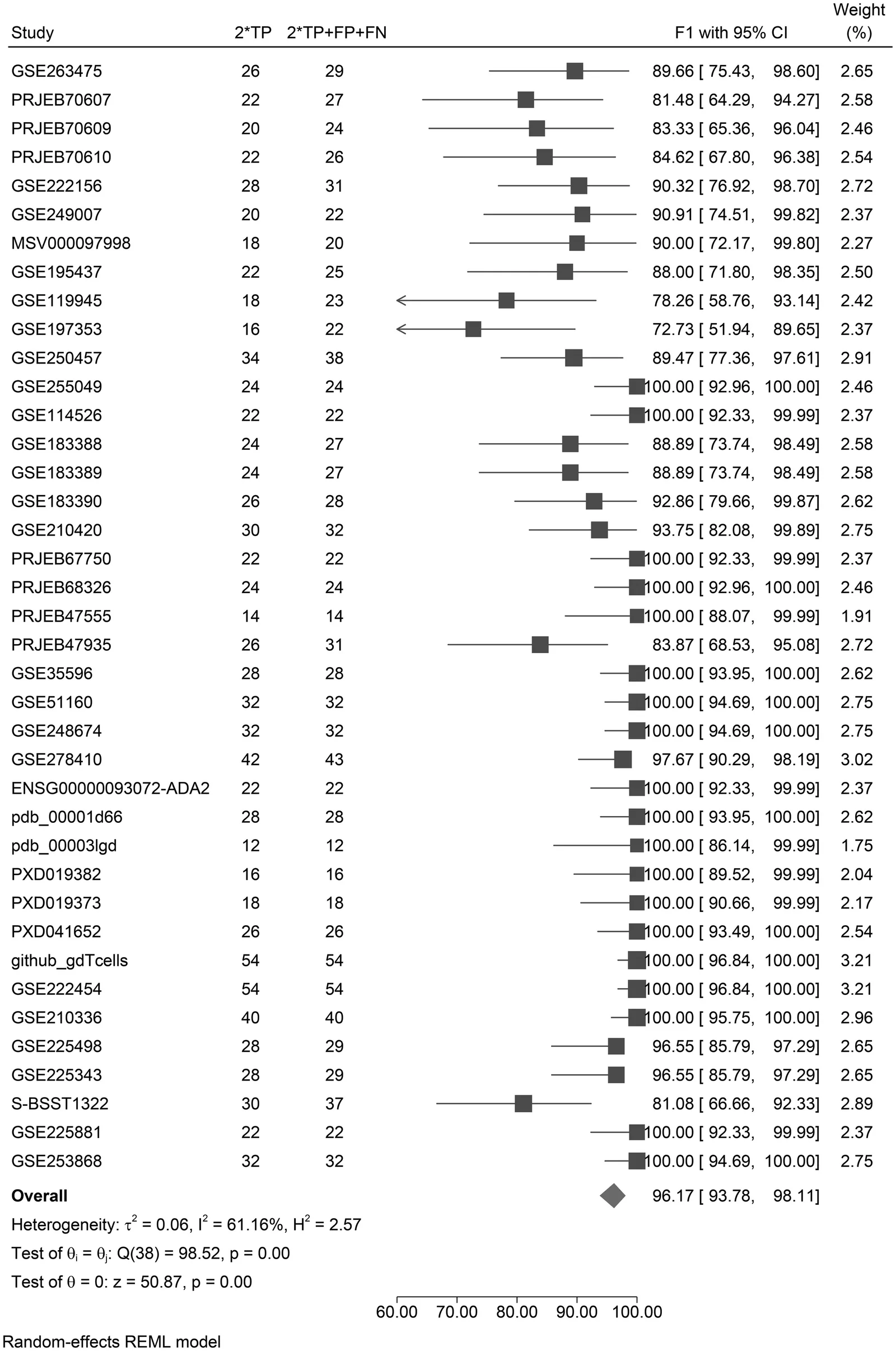
F1 Score of the multi-step LLM workflow according to the human-in-the-loop verification of LLM suggestions.

## 4 Discussion

In the initial evaluation of the LLM suggestions for 39 distinct datasets, the workflow maintained robust performance comparable to the exploratory phase. Preliminary analysis of user interactions reveals that the Gemini 3 Pro model achieved an aggregate precision of approximately 99.65%, meaning authors accepted the vast majority of pre-filled fields without modification. By contrast, the recall was lower at roughly 93.75%. This gap reflects instances where the model missed entities that weren’t explicitly listed on the repository landing page, requiring authors to manually add specific details. Therefore, while the high precision ensured that authors rarely had to delete incorrect suggestions (false positives), the recall metrics show that human expertise is still necessary to catch missing information (false negatives). Ultimately, the workflow successfully shifted the user’s role from drafting to reviewing, significantly reducing the time required for metadata documentation while ensuring high schema compliance.

These estimates require careful interpretation. Because authors judged the pre-filled fields only after seeing them, acceptance cannot be equated with correctness: plausible but incorrect entries may have been retained and counted as true positives, and omissions that an anchored reader does not notice are not recorded as false negatives. The author-verified metrics therefore describe a best case. To bound performance from the opposite side, Supplementary Appendix 2, available at supplementary material  *Bioinformatics Advances* online reports an independent evaluation against the annotations recorded during the schema-development process (Engel *et al.* 2026), adjudicated by the research data management team without re-consulting the original scientists, so that every deviation from the reference–including defensible near-synonyms and alternative levels of granularity–is charged as an error. Under that deliberately unforgiving rule, pooled F1 across six current models ranges from 53.0% to 66.5%. Neither figure is the truth: the author-verified analysis maximises true positives while minimising both error types, and the reference-based analysis maximises both error types. The realistic operating point lies between them, and we report the bracket rather than a single point estimate.

Integrating this AI workflow into fredato illustrates the practical benefits of a hybrid approach. By pre-filling identifiers and entity tags, the model addresses the “blank slate” issue, reducing the workload for researchers authoring multiple records (Wang *et al.* 2024). Crucially, the workflow maintains data integrity by requiring scientists to review the output, providing a checkpoint to correct errors or nuances the model may miss (Reddy 2024, Wang *et al.* 2024, Kaier *et al.* 2026). This approach balances automation with expert verification, compressing handling time while capturing necessary corrections (Wang *et al.* 2024).

The technically forced human-in-the-loop design not only represents a significant step in addressing the “metadata bottleneck” (Engel *et al.* 2026, Wood *et al.* 2026), but also follows established frameworks for responsible automation, positioning artificial intelligence as a context-sensitive amplifier intended to support rather than supplant human expertise (Reddy 2024, Wood *et al.* 2026). Such structured interactions are critical safeguards against model hallucinations, ensuring that high-stakes research data remain trustworthy and auditable (Mortezaagha *et al.* 2025, Wood *et al.* 2026).

The use of a highly granular, hierarchical metadata schema is instrumental to this process, as it acts as a semantic backbone that constrains model inference through typed fields and controlled vocabularies (Mortezaagha *et al.* 2025, Engel *et al.* 2026). Our results support the paradigm shift where schemas evolve from static data entry forms into high-dimensional **“**instruction sets**”** for agentic workflows (Engel *et al.* 2026). In this model, the AI does not merely extract text but actively validates its decisions against schema constraints in real-time (Engel *et al.* 2026). Furthermore, reformatting complex metadata dictionaries into structured JSON schemas has been shown to significantly improve a model’s ability to correctly process and extract numerous features simultaneously (Balasubramanian *et al.* 2025). These schema-based predictions allow for high-accuracy concept extraction that was previously unfeasible in purely manual routines (Ikeda *et al.* 2025).

Regarding model selection, while the debate between general-purpose and specialized LLMs remains nuanced, our findings suggest that generalist models like Gemini, when properly grounded through appropriate data, can achieve performance comparable to trained human annotators (Balasubramanian *et al.* 2025, Wood *et al.* 2026). A head-to-head comparison of six current models from four providers under an identical pipeline and an identical reference standard is reported in Supplementary Appendix 2, available at supplementary material  *Bioinformatics Advances* online. Pooled F1 ranged from 53.0% to 66.5%, with five of the six models within a 5.5 percentage-point band, and the cost-optimised models performing on a par with the frontier models. A comparison against dedicated biomedical named-entity recognition tools such as PubTator, SciSpacy or BioBERT-derived taggers was not performed, because these emit entities from general biomedical vocabularies rather than from a hierarchical, consortium-specific controlled vocabulary with conditional item logic; scoring them against the present reference standard would require an intermediate mapping layer whose quality would dominate the measured performance. Notably, the open-weight model included in the comparison achieved the highest pooled precision of the six models evaluated, so the workflow is not dependent on a proprietary model and an open-weight substitute can be deployed without redesign. While domain-specific fine-tuning often yields superior accuracy on niche terminology, generalist foundation models inherit strong generalization capabilities that are frequently lost in over-specialized architectures (Balasubramanian *et al.* 2025, Verbitsky *et al.* 2025) The high precision (99.65%) achieved in our pilot is vital for knowledge discovery, as it minimizes the risk of misleading hypotheses (Brokman *et al.* 2025). However, the moderate recall (93.75%) highlights a persistent challenge where models may overlook entities in documents with high information density, reinforcing the necessity for expert post-validation to capture missing entries (Pangakis *et al.* 2023, Wang *et al.* 2024, Brokman *et al.* 2025).

A workflow built on a single proprietary model raises the legitimate concern that results obtained with one release may not survive the next. Supplementary Appendix 2, available at supplementary material  *Bioinformatics Advances* online addresses this directly. Six models from four providers, spanning frontier and cost-optimized tiers, were run through the identical pipeline without any adaptation of prompts, schema representation or post-processing, and all six produced usable, schema-conformant annotations. Five of the six produced pooled F1 values within a band of 5.5 percentage points (61.0%–66.5%); only one model fell clearly outside it. The operating characteristics of this workflow are therefore determined mainly by the orchestration–two-step prompting, landing-page grounding, schema flattening, strict JSON remapping and provenance tracking–rather than by the identity of the underlying model, and model substitution is possible without redesign. Notably, the cost-optimized models were not inferior to the frontier models on this task, suggesting that grounded extraction against a controlled vocabulary is constrained more by what the repository landing page actually states than by the reasoning capacity of the model, with corresponding consequences for the cost of operating such a pipeline at consortium scale.

Despite these promising results, significant limitations exist regarding the isolated situation within a CRC. The schemas utilized are often highly granular but specific to individual research centers, which can lead to limited vocabulary overlap and potential friction when datasets deviate from the consortium’s primary focus (Engel *et al.* 2026, Kaier *et al.* 2026). Such isolation runs the risk of creating siloed datasets that remain difficult to harmonize across broader institutional networks. Finally, the external validity of this pilot is constrained by its relatively small and homogeneous sample size (Jabal *et al.* 2024, Mortezaagha *et al.* 2025). To ensure the long-term utility and safe adoption of these AI-augmented routines, broader validation across multi-site cohorts and more diverse scientific domains is required to account for the heterogeneous reporting practices found across the wider biomedical literature (Artsi *et al.* 2025, Mortezaagha *et al.* 2025).

The practical value of higher metadata completeness lies downstream of annotation. The schema underlying this workflow was designed as a consortium-wide discovery and reuse layer rather than as an administrative record, with curated levels mapped to established ontologies such as EFO, the BRENDA Tissue Ontology and Cellosaurus, and with organising principles for knowledge-graph construction published alongside the schema configurations (Engel *et al.* 2026). Pre-annotation raises the fill rate of precisely those retrieval dimensions–organism, developmental timeline, tissue source, cell line, intervention and readout–on which structured dataset queries and cross-study integration depend, and which are most frequently left empty under purely manual annotation. Because every entry carries a provenance tag recording its origin and validation status, downstream consumers can filter or weight machine-suggested and human-verified entries differently, which is a prerequisite for using such records in automated integration or knowledge-graph pipelines. We have not attempted to quantify the retrieval benefit experimentally, as this would require a query workload and relevance judgements that constitute a separate study.

One of the primary concerns identified in recent pilots is the non-deterministic nature of Large Language Models (LLMs), which produce variable outputs that are highly version-dependent (Giuliani *et al.* 2025, Kaier *et al.* 2026, Watter *et al.* 2026). This lack of deterministic consistency makes exact replication of metadata extraction results difficult (Kaier *et al.* 2026, Watter *et al.* 2026). Pinning model version, prompt, temperature and seed constrains, but does not eliminate, this variability, because provider-side routing, time-dependent retrieval of live landing pages and batch-dependent inference remain outside the caller’s control. We therefore quantified the residual variability directly: across five repeated executions on identical inputs, the mean pairwise Jaccard index of the resulting annotation sets ranged from 0.784 to 0.908 across six current models (Supplementary Appendix 2, Table S8, available at supplementary material  *Bioinformatics Advances* online). Repeated execution thus reproduces roughly four fifths to nine tenths of the annotation set, depending on the model. We suggest that reproducibility for this class of workflow be reported as a measured consistency band alongside the model, the provider and the retrieval date, and that annotation output be treated as a versioned artefact with recorded provenance–which is the purpose of the provenance tagging described in the Supplementary Appendix 2, available at supplementary material  *Bioinformatics Advances* online. Additionally, the “black box” phenomenon of AI reasoning continues to impact trust; without transparent and explainable decision pathways, expert clinicians and researchers may be hesitant to rely on AI-generated metadata for high-stakes evidence synthesis (Hassan *et al.* 2024, Trinkley *et al.* 2024, Anthuvan *et al.* 2025). Establishing robust post-deployment monitoring is therefore essential to detect model drift and ensure that the AI continues to perform safely and accurately as documentation practices evolve (Artsi *et al.* 2025). Unfortunately, the present sample also does not support a breakdown of recall by schema field type. With 324 curated levels distributed over some 30 schema items and 39 evaluated datasets, most items are populated in only a handful of datasets, so per-item estimates carry confidence intervals spanning most of the unit interval. Identifying which metadata categories the pipeline handles least reliably is a well-defined objective for a larger, multi-consortium sample.

## Supplementary Material

vbag283_Supplementary_Data

## Data Availability

The metadata schema underpinning the annotation workflow has been published in full on FreiData and is openly accessible (DOI: 10.60493/n9sbn-5ww23). The aggregated performance data (true positives, false positives, and false negatives per verified dataset) on which all meta-analytic estimates are based are presented in full in Figs 3–5 of this manuscript and are therefore directly available to readers without restriction. No additional datasets were generated that are not represented in these figures.
