# Supplementary material for: Accelerating metadata annotation in collaborative research centers: a hybrid AI workflow for biomedical entities
Source: Bioinform Adv. 2026 Sep 23;6(1):vbag283. doi: 10.1093/bioadv/vbag283 (PMC13690486; doi:10.1093/bioadv/vbag283)
Supplement: Supplementary file 1 [file vbag283_supplementary_data.zip › 20260819_Supplementary Appendix 2.docx]

Supplementary Appendix 2

Benchmarking against schema-development annotations: a worst-case evaluation and repeated-run consistency of six large language models

Watter M, Engel F, Kalantari A, Giuliani C, Schuller K, Franzke C-W, Sperandio M, Binder H, Kaier K

*Supplement to:* Accelerating metadata annotation in collaborative research centers: A hybrid AI workflow for biomedical entities

# Rationale

The evaluation reported in the main manuscript maps author interactions in the fredato validation interface onto performance metrics: accepted suggestions count as true positives (TP), deleted suggestions as false positives (FP), and manually added entities as false negatives (FN). This design has two structural weaknesses. First, authors form their judgment only after seeing the pre-filled LLM output, so acceptance is confounded by anchoring and by a default-acceptance tendency; plausible-looking but incorrect entries may be retained and counted as TP rather than FP. Second, an FN is recorded only when an author proactively adds a missing entity, so omissions that an author anchored by the pre-filled form does not notice are systematically undercounted. Both mechanisms push the estimates upward. The precision of 99.65% and the recall of 93.75% reported in the main manuscript should therefore be read as an upper bound on what the workflow achieves under favorable conditions, rather than as an unbiased estimate of extraction accuracy.

The methodologically clean remedy would be an independent, blinded and well-instructed expert annotation produced by annotators who never see the LLM output. In this setting that is not realistically achievable. During the development of the PILOT metadata schema we conducted guided trial annotations with researchers from the consortium (Engel et al., 2026a). These sessions were supervised by research data managers, and because participants had volunteered and had a strong intrinsic interest in seeing every aspect of their own work represented in the schema, they invested the time required to annotate their datasets thoroughly. Even under these favorable conditions the annotations tended to sprawl as soon as strict guidance was relaxed, and considerable moderation was needed to keep them within the intended scope. In routine operation this level of engagement cannot be assumed. Annotation competes with immediate research demands for available time, participation is voluntary, and the typical failure mode can be expected to be not over-annotation but sparse annotation. A prospective blinded double annotation performed under everyday conditions would, therefore, either not be completed at all or would itself be of unknown and probably poor quality — and would then serve as a gold standard no more trustworthy than the one it is meant to replace.

We therefore implemented a compromise that brackets the true performance from the opposite side. The annotations produced during the schema-development process were adopted as a fixed gold standard, and the datasets underlying those annotations were re-annotated by the current workflow. The reconciliation of model output against this gold standard was carried out exclusively by the research data management team; the original scientists were not consulted again. As a consequence, every model suggestion that does not correspond exactly to a term selected during schema development is counted as a false positive, even where it is defensible or arguably correct, and every gold term not reproduced by the model is counted as a false negative, even where the model selected a near-synonym or a different level of granularity. This is an unforgiving rule, and it is intended to be. The resulting metrics constitute a lower bound — a worst-case scenario for LLM-based annotation. Taken together with the main analysis, the two evaluations bracket the plausible operating range of the workflow rather than estimating a single point within it.

Because this evaluation requires no interaction with the original authors, it can be repeated at will. We used this property to address two further points. First, Gemini 3.0 Pro, the model used in the main analysis, has since been superseded; we, therefore, re-ran the identical pipeline with six current models from four providers. Second, for each of these models we executed five independent repetitions on identical inputs, which allows the run-to-run consistency of a non-deterministic pipeline to be quantified empirically rather than asserted.

# Methods

## Gold standard and evaluation corpus

The gold standard consists of the dataset annotations recorded by researchers during the guided trial annotations of the PILOT schema-development process (Engel et al., 2026a). These annotations were produced through the fredato web interface before any LLM output existed for the corresponding datasets, and they are, therefore, not biased by the pre-annotation being evaluated here. They exist for 12 public dataset identifiers. Datasets were matched to model output by repository accession.

Annotations entered as free text were excluded from the comparison. The schema occasionally permits free-text entries where a required keyword is not available for selection, but the models were not tasked with proposing new keywords, and a free-text gold value can neither be matched nor fairly counted as a miss. One such manually entered term (Macrophage progenitors) was removed on these grounds. Controlled parent levels that had been selected separately alongside a free-text child remain counted. The exclusion of free-text entries was the only annotation decision changed relative to the original git-tracked audit record; the results reported here were recalculated from that audit so that this change is fully traceable.

For the three public accessions listed in the baseline manifest under a combined European Nucleotide Archive submission, the corresponding combined manual annotation was applied to each accession.

## Schema coverage and counting rules

The study used version 1.0.0 of the PILOT metadata schema as documented in the schema publication (Engel et al., 2026b). The trial annotations used for the gold standard were produced with a schema prototype that differed slightly from the first production version. Any data items contained in both versions are covered here. Items from version 1.0.0 are referenced by their schema ID; prototype-only items are referenced by name alone.

The PILOT schema is hierarchical. Some items are always available, whereas others become visible only when a relevant option has been selected in a parent item. We therefore distinguish always-available top-level items from conditional dependent items. The following top-level items were evaluated:

Table S1. Top-level schema items included in the comparison.

| ID | Data item |
| --- | --- |
| 14 | Data source name |
| 15 | Dataset content description |
| 16 | Is the data published? |
| 26 | Organism |
| 34 | Intervention |
| 40 | Sample preparation |
| 42 | Sample processing |
| 43 | Readout |

The following dependent items were evaluated whenever the condition given in the right-hand column was met by a rating of a top-level item:

Table S2. First-order dependent schema items and their display conditions.

| ID | Data item | Condition |
| --- | --- | --- |
| 17 | Storage location | 16 Is the data published? == NO |
| 19 | Responsible contact | 16 Is the data published? == NO |
| 20 | Storage details | 16 Is the data published? == NO |
| 21 | Publication service | 16 Is the data published? == YES |
| 22 | Comment | 16 Is the data published? == YES |
| 23 | Identifier | 16 Is the data published? == YES |
| 27 | Time line | 26 Organism NOT EMPTY |
| 28 | Mouse line | 26 Organism == 26.2 Mouse |
| 29 | Tissue source | 26 Organism NOT EMPTY |
| 33 | Cell line | 26 Organism NOT EMPTY |
| 35 | Genes of interest | 34 Intervention == 34.1 Genetic modification |
| 36 | Pharmacological/dietary interventions | 34 Intervention == 34.2 Pharmacological treatment |
| 37 | Infection | 34 Intervention == 34.3 Infection |
| 38 | Injection | 34 Intervention == 34.4 Injection |
| 39 | Housing condition | 34 Intervention == 34.5 Housing condition |
| 41 | Cell type | 40 Sample preparation == 40.1 Cultured cells OR 40.2 Isolated cells |
| 44 | Biomechanics | 43 Readout == 43.1 Biomechanics |
| 45 | Clinical monitoring | 43 Readout == 43.2 Clinical monitoring |
| 47 | Light microscopy | 43 Readout == 43.6 Light microscopy |
| 48 | Cellular/molecular biology | 43 Readout == 43.7 Cellular/molecular biology |
| 49 | Sequencing | 43 Readout == 43.10 Sequencing |

The following items were evaluated whenever their condition was met by a rating of another dependent item:

Table S3. Second-order dependent schema items and their display conditions.

| ID | Data item | Condition |
| --- | --- | --- |
| 24 | DOI | 23 Identifier == “DOI” |
| 25 | URL | 23 Identifier == “URL” |
| 30 | Blood | 29 Tissue source == 29.1 Blood |
| 31 | Part of intestine | 29 Tissue source == 29.4 Intestine |
| 32 | Area of lymph node | 29 Tissue source == 29.9 Lymph node |
| — | Skin | 29 Tissue source == 29.11 Skin |
| — | Vascular system | 29 Tissue source == 29.14 Vascular system |
| — | CNS | 29 Tissue source == 29.16 CNS |

**Counting of annotation nodes.** Scientists annotating through the fredato interface cannot rate a dependent item unless the conditions for its display are met, so certain entries in top-level or intermediate items are structurally required before the dependent item becomes available. The models, by contrast, were prompted with a flattened representation of the schema (Appendix S3) and could therefore rate a dependent item even where the licensing parent had not been selected.

To establish the count of annotations, all selected levels were counted. Where both a dependent item and the item on which it depends were rated, levels from both were counted. Where only the dependent item was rated, the levels selected there were still counted, but the upper-level item was not added, even where its rating was implied. For example, if the model proposed level 43.6 Light microscopy for item 43 Readout and the levels 47.1 Confocal and 47.5 Fluorescence for item 47 Light microscopy, the total count is three; if only the two levels of item 47 were proposed, the count is two.

**Error definitions.** Each selected level of a hierarchical annotation was counted as a separate annotation node. Proposed and gold annotation nodes were compared one occurrence at a time. Each proposed occurrence that matched the same node in the gold annotation was counted as one true positive. Unmatched proposed occurrences were counted as false positives, while unmatched gold occurrences were counted as false negatives. If the gold annotation contained both a parent and a child, both were counted separately. If the model proposed the parent but not the child, the parent was counted as a true positive and the child as a false negative. If neither was proposed, both were counted as false negatives. Repeated occurrences were matched and counted separately.

Example 1: suppose the gold annotation contains Mouse in the Organism field and Sequencing > Single-cell RNA sequencing in the Readout field. Here, Readout is the metadata field, Sequencing is the parent node, and Single-cell RNA sequencing is the child node. The model instead proposes Mouse and Sequencing > Spatial Transcriptomics, where Spatial Transcriptomics is another child node of Sequencing. Mouse and the shared parent Sequencing are true positives, Spatial Transcriptomics is a false positive, and Single-cell RNA sequencing is a false negative. Good LLM performance means finding many gold nodes while proposing few additional nodes and missing few gold nodes.

Example 2: suppose the gold annotation contains Mouse in the Organism field and Sequencing > Single-cell RNA sequencing in the Readout field, while the model proposes only Mouse and provides no annotation for the Readout field. Mouse is one true positive. Because neither the parent node Sequencing nor the child node Single-cell RNA sequencing was proposed, both are false negatives. The result is therefore one true positive, no false positives, and two false negatives.

## Models and inference parameters

Six models were evaluated through the identical pipeline, without any model-specific adaptation of prompts, schema representation or post-processing:

Table S4. Models evaluated in the supplementary benchmark.

| Display name | Provider |
| --- | --- |
| Claude Opus 5 | Anthropic |
| GPT-5.6-sol | OpenAI |
| Gemini 3 Flash | Google |
| Gemini 3.1 Pro | Google |
| Gemini 3.6 Flash | Google |
| GLM-5.2 | Z.ai (Zhipu AI) |

All models were accessed through the OpenRouter API using the documented provider defaults. Sampling parameters were not pinned: temperature, top_p and seed were left at their defaults rather than being set to temperature 0 with a fixed seed. This was a deliberate choice. The workflow is intended to reflect realistic deployment conditions, and — more importantly — pinning these parameters would not in fact guarantee reproducible output, because several sources of non-determinism lie outside the caller’s control:

1. **Provider-side load management.** Providers may silently route requests to quantized or otherwise substituted variants of a model under load. This is not visible in the response payload.
2. **Time-dependent retrieval.** The pipeline renders repository landing pages at request time. Page content can carry volatile elements that change on every visit; NCBI GEO pages, for example, embed a per-request session identifier (<meta name="ncbi_sessionid" …>) that enters the prompt together with the remaining page text and can propagate into all downstream output.
3. **Batch-dependent inference.** Requests processed concurrently within the same inference batch can alter normalization behavior in certain model layers, so output may depend on unrelated load present at the moment of the call (Thinking Machines Lab, 2025).
4. **Router behavior** Router metadata was not yet exposed by OpenRouter at the time of the experiments. Because a single provider and a single model were configured for each run, routing should not have contributed additional variance, but this could not be verified from the returned payloads.

We, therefore, regard the empirical characterization of run-to-run variability as more informative than a nominal parameter setting that cannot be enforced end to end. The practical implication is that reproducibility of this class of workflow has to be reported as a measured consistency band, not as a configuration flag.

## Repeated-run consistency

Each model was executed five times on the identical input set. For every pair of runs and every gold dataset detected in both runs of that pair, the Jaccard index of the two resulting sets of annotation nodes was computed as

*J* = |*A* ∩ *B*| / |*A* ∪ *B*|,

and the values were averaged across dataset and run pairs to yield one consistency value per model. A value of 1 indicates that the model returned exactly the same annotation set in every repetition; lower values indicate that repeated calls on identical input produced diverging annotations.

Entity-level precision, recall and F1 were derived exclusively from the first of the five runs, so that the accuracy estimates describe a single realistic pipeline execution rather than an average over repetitions.

## Statistical analysis

The statistical treatment is identical to that of the main manuscript. For each model and each dataset, the three metrics were treated as single proportions:

Precision = TP / (TP + FP); Recall = TP / (TP + FN); F1 = 2·TP / (2·TP + FP + FN).

Because the per-dataset denominators are small and several proportions lie close to the boundary of the parameter space, the Freeman–Tukey double arcsine transformation was applied to stabilize the variance (Miller, 1978). Aggregate estimates and 95% confidence intervals were obtained from a random-effects model using the restricted maximum likelihood (REML) estimator and back-transformed using the harmonic mean of the sample sizes (Schwarzer & Rücker, 2022). Heterogeneity is reported as τ², I² and Cochran’s Q. Analyses were performed in Stata using meta esize, meta summarize and meta forestplot. Each of the 18 analyses (six models × three metrics) is based on the same 10 datasets.

# Results

## Dataset detection and evaluation corpus

Of the 12 public dataset identifiers listed in the gold manifest, 10 were detected by the models and could be matched to a gold annotation; 2 were not detected. The 10 matched datasets originate from three publications (PMIDs 36633909, 39899882 and 40934928). A fourth publication carrying public gold identifiers contributed no matched dataset and therefore does not enter the entity metrics. Dataset detection is evaluated separately and is not folded into the precision, recall and F1 values reported below; the entity metrics are conditional on detection having succeeded. One discrepancy between the accessions GSE250456 and GSE250457 remains unresolved and awaits manual adjudication; it is excluded from the analysis.

Across the 10 matched datasets the gold standard comprises 105 annotation nodes, ranging from 6 to 16 nodes per dataset. Per-dataset counts of true positives, false positives and false negatives for all six models are given in Table S5.

Table S5. True positives / false positives / false negatives per dataset and model, first run. Gold nodes = TP + FN.

| Dataset | Gold nodes | Claude Opus 5 | GLM-5.2 | GPT-5.6-sol | Gemini 3 Flash | Gemini 3.1 Pro | Gemini 3.6 Flash |
| --- | --- | --- | --- | --- | --- | --- | --- |
| GSE119945 | 8 | 5 / 5 / 3 | 4 / 2 / 4 | 5 / 7 / 3 | 5 / 2 / 3 | 4 / 1 / 4 | 4 / 1 / 4 |
| GSE195437 | 8 | 5 / 8 / 3 | 5 / 1 / 3 | 5 / 8 / 3 | 5 / 2 / 3 | 5 / 2 / 3 | 5 / 1 / 3 |
| GSE197353 | 8 | 6 / 5 / 2 | 5 / 1 / 3 | 6 / 5 / 2 | 6 / 3 / 2 | 5 / 0 / 3 | 5 / 0 / 3 |
| GSE249007 | 8 | 6 / 6 / 2 | 5 / 2 / 3 | 6 / 7 / 2 | 5 / 3 / 3 | 5 / 2 / 3 | 5 / 2 / 3 |
| MSV000097998 | 6 | 5 / 3 / 1 | 4 / 3 / 2 | 5 / 4 / 1 | 4 / 4 / 2 | 3 / 5 / 3 | 4 / 3 / 2 |
| GSE263475 | 16 | 14 / 15 / 2 | 12 / 11 / 4 | 13 / 14 / 3 | 13 / 10 / 3 | 7 / 9 / 9 | 13 / 10 / 3 |
| PRJEB70607 | 14 | 12 / 9 / 2 | 11 / 10 / 3 | 12 / 9 / 2 | 12 / 9 / 2 | 7 / 10 / 7 | 12 / 9 / 2 |
| PRJEB70609 | 14 | 12 / 6 / 2 | 11 / 5 / 3 | 12 / 5 / 2 | 12 / 4 / 2 | 7 / 5 / 7 | 12 / 4 / 2 |
| PRJEB70610 | 14 | 12 / 12 / 2 | 11 / 5 / 3 | 12 / 12 / 2 | 12 / 9 / 2 | 7 / 5 / 7 | 12 / 12 / 2 |
| GSE222156 | 9 | 5 / 9 / 4 | 4 / 4 / 5 | 5 / 9 / 4 | 5 / 9 / 4 | 5 / 9 / 4 | 5 / 9 / 4 |
| **Total** | **105** | **82 / 78 / 23** | **72 / 44 / 33** | **81 / 80 / 24** | **79 / 55 / 26** | **55 / 48 / 50** | **77 / 51 / 28** |

## S7.2 Entity-level performance

Under this deliberately strict gold standard, all six models perform far below the values obtained in the author-verified main analysis. Pooled precision ranged from 50.4% to 62.6%, recall from 52.4% to 79.2%, and F1 from 53.0% to 66.5% (Table S6).

Table S6. Random-effects pooled estimates (%) across 10 datasets, Freeman–Tukey double arcsine transformation, REML. Corresponding forest plots are given in Supplemental Figures 2–19.

| Model | Precision (95% CI) | Recall (95% CI) | F1 (95% CI) |
| --- | --- | --- | --- |
| Claude Opus 5 | 51.28 (43.13–59.40) | 79.17 (70.04–87.23) | 62.13 (56.01–68.07) |
| GLM-5.2 | 62.56 (52.81–71.90) | 69.26 (59.36–78.46) | 65.45 (58.78–71.85) |
| GPT-5.6-sol | 50.36 (42.27–58.45) | 78.08 (68.84–86.29) | 61.02 (54.46–67.40) |
| Gemini 3 Flash | 59.22 (50.22–67.96) | 76.29 (66.88–84.73) | 66.46 (60.11–72.55) |
| Gemini 3.1 Pro | 55.55 (43.45–67.37) | 52.38 (42.13–62.55) | 52.97 (45.91–59.98) |
| Gemini 3.6 Flash | 62.43 (51.49–72.85) | 74.54 (64.99–83.20) | 66.49 (59.93–72.78) |

Heterogeneity was negligible in most analyses. I² was 0.00% in 14 of the 18 analyses and did not exceed 22.9% in any of them; Cochran’s Q was non-significant throughout (all p ≥ 0.15). The models therefore performed similarly across datasets rather than failing on isolated cases — the reduction relative to the main analysis is systematic, not driven by outliers. Detailed heterogeneity statistics are given in Table S7.

Table S7. Heterogeneity statistics for all 18 random-effects analyses.

| Model | Metric | τ² | I² (%) | Q (df = 9) | p |
| --- | --- | --- | --- | --- | --- |
| Claude Opus 5 | Precision | 0.0000 | 0.00 | 4.47 | 0.878 |
| Claude Opus 5 | Recall | 0.0000 | 0.00 | 6.24 | 0.716 |
| Claude Opus 5 | F1 | 0.0000 | 0.00 | 8.62 | 0.473 |
| GLM-5.2 | Precision | 0.0000 | 0.00 | 4.85 | 0.847 |
| GLM-5.2 | Recall | 0.0000 | 0.00 | 5.71 | 0.769 |
| GLM-5.2 | F1 | 0.0000 | 0.00 | 5.11 | 0.825 |
| GPT-5.6-sol | Precision | 0.0000 | 0.00 | 5.47 | 0.791 |
| GPT-5.6-sol | Recall | 0.0000 | 0.00 | 5.41 | 0.797 |
| GPT-5.6-sol | F1 | 0.0050 | 11.83 | 10.14 | 0.339 |
| Gemini 3 Flash | Precision | 0.0000 | 0.00 | 5.77 | 0.762 |
| Gemini 3 Flash | Recall | 0.0000 | 0.00 | 6.37 | 0.702 |
| Gemini 3 Flash | F1 | 0.0000 | 0.00 | 8.31 | 0.503 |
| Gemini 3.1 Pro | Precision | 0.0280 | 22.88 | 13.30 | 0.150 |
| Gemini 3.1 Pro | Recall | 0.0000 | 0.00 | 1.42 | 0.998 |
| Gemini 3.1 Pro | F1 | 0.0000 | 0.00 | 8.51 | 0.484 |
| Gemini 3.6 Flash | Precision | 0.0231 | 22.86 | 12.83 | 0.170 |
| Gemini 3.6 Flash | Recall | 0.0000 | 0.00 | 8.34 | 0.500 |
| Gemini 3.6 Flash | F1 | 0.0012 | 2.63 | 8.63 | 0.472 |

Gemini 3.6 Flash (F1 66.49%, 95% CI 59.93–72.78) and Gemini 3 Flash (66.46%, 60.11–72.55) achieved the highest combined performance, closely followed by GLM-5.2 (65.45%, 58.78–71.85). Claude Opus 5 achieved the highest recall (79.17%, 70.04–87.23) but, together with GPT-5.6-sol, the lowest precision (51.28%, 43.13–59.40 and 50.36%, 42.27–58.45, respectively). Gemini 3.1 Pro was the weakest model overall, and the only one whose recall fell below 60% (52.38%, 42.13–62.55; F1 52.97%, 45.91–59.98).

The precision–recall pattern tracks the number of annotation nodes each model proposes. Against the 105 gold nodes, GPT-5.6-sol proposed 161 nodes and Claude Opus 5 proposed 160, followed by Gemini 3 Flash (134), Gemini 3.6 Flash (128), GLM-5.2 (116) and Gemini 3.1 Pro (103). Verbose models recover more of the gold terms but accumulate more non-matching suggestions, and under the present rule every non-matching suggestion is charged as an error irrespective of whether a domain expert would have accepted it. The precision figures in Table S6 should therefore not be read as a hallucination rate. They quantify divergence from one specific, unadjudicated reference annotation.

## S7.3 Repeated-run consistency

The mean pairwise Jaccard index across the five repetitions ranged from 0.784 to 0.908 (Table S8).

Table S8. Within-model annotation consistency across five repeated runs on identical inputs, averaged over run pairs and the 10 matched gold datasets. Higher values indicate more similar annotations across repetitions.

| Model | Mean Jaccard index |
| --- | --- |
| Claude Opus 5 | 0.908 |
| GPT-5.6-sol | 0.836 |
| Gemini 3.6 Flash | 0.822 |
| Gemini 3.1 Pro | 0.799 |
| GLM-5.2 | 0.785 |
| Gemini 3 Flash | 0.784 |

Claude Opus 5 was the most stable model, reproducing roughly nine tenths of its own annotation set on identical input, while Gemini 3 Flash and GLM-5.2 reproduced approximately four fifths. Run-to-run variability is therefore real, but it is bounded, and it is small relative to the gap between the best-case and worst-case accuracy estimates. Consistency and accuracy were not aligned: the most reproducible model had the lowest precision, and the two models with the highest F1 were mid-range in consistency. Reproducibility of output and correctness of output are distinct properties of a pipeline, and neither can substitute for the other. For a human-in-the-loop workflow this matters in a specific way — a model that is stable but systematically verbose imposes a predictable, and therefore manageable, review burden, whereas an unstable model makes the review burden itself unpredictable.

## S7.4 Forest plots

Supplemental Figures 2–19 present the dataset-level estimates and the pooled random-effects estimate for each model and metric. Figures 2–7 show precision, Figures 8–13 recall, and Figures 14–19 the F1 score.

### Precision

Supplemental Figure 2: Precision of the annotation workflow using Claude Opus 5, benchmarked against the schema-development gold standard

Supplemental Figure 3: Precision of the annotation workflow using GLM-5.2, benchmarked against the schema-development gold standard

Supplemental Figure 4: Precision of the annotation workflow using GPT-5.6-sol, benchmarked against the schema-development gold standard

Supplemental Figure 5: Precision of the annotation workflow using Gemini 3 Flash, benchmarked against the schema-development gold standard

Supplemental Figure 6: Precision of the annotation workflow using Gemini 3.1 Pro, benchmarked against the schema-development gold standard

Supplemental Figure 7: Precision of the annotation workflow using Gemini 3.6 Flash, benchmarked against the schema-development gold standard

### Recall

Supplemental Figure 8: Recall of the annotation workflow using Claude Opus 5, benchmarked against the schema-development gold standard

Supplemental Figure 9: Recall of the annotation workflow using GLM-5.2, benchmarked against the schema-development gold standard

Supplemental Figure 10: Recall of the annotation workflow using GPT-5.6-sol, benchmarked against the schema-development gold standard

Supplemental Figure 11: Recall of the annotation workflow using Gemini 3 Flash, benchmarked against the schema-development gold standard

Supplemental Figure 12: Recall of the annotation workflow using Gemini 3.1 Pro, benchmarked against the schema-development gold standard

Supplemental Figure 13: Recall of the annotation workflow using Gemini 3.6 Flash, benchmarked against the schema-development gold standard

### F1 score

Supplemental Figure 14: F1 score of the annotation workflow using Claude Opus 5, benchmarked against the schema-development gold standard

Supplemental Figure 15: F1 score of the annotation workflow using GLM-5.2, benchmarked against the schema-development gold standard

Supplemental Figure 16: F1 score of the annotation workflow using GPT-5.6-sol, benchmarked against the schema-development gold standard

Supplemental Figure 17: F1 score of the annotation workflow using Gemini 3 Flash, benchmarked against the schema-development gold standard

Supplemental Figure 18: F1 score of the annotation workflow using Gemini 3.1 Pro, benchmarked against the schema-development gold standard

Supplemental Figure 19: F1 score of the annotation workflow using Gemini 3.6 Flash, benchmarked against the schema-development gold standard

# Discussion

## Bracketing rather than estimating

The two evaluations bound the performance of the workflow rather than estimating it. The author-verified analysis in the main manuscript assumes that everything an author accepted was correct and that everything an author did not add was absent from the ground truth. This maximizes true positives while minimizing false positives and false negatives, and yields precision 99.65%, recall 93.75% and F1 96.17%. The present analysis assumes that only the exact terms recorded during the schema-development process are correct, adjudicated by research data managers without consulting the original scientists. This maximizes both false positives and false negatives, and yields F1 values between 52.97% and 66.49%.

Neither figure is the truth. The realistic operating point lies between them, and the width of that interval is the honest summary of what a human-in-the-loop pre-annotation pilot of this size can establish. We consider it more useful to state this bracket explicitly than to defend a single point estimate obtained under a design that cannot separate acceptance from correctness.

Several features of the present design push the lower bound below the plausible true value, and it is worth naming them so that the bound is not over-interpreted:

- **No adjudication of near-misses.** Genuine synonyms, defensible alternative granularities and terms that a domain expert would have endorsed are all charged as errors, because the original scientists were not re-consulted. This is the single largest source of pessimism in the estimate.
- **Interface asymmetry.** The gold annotations were produced through the fredato interface, which enforces the conditional display logic of the hierarchical schema. The models were prompted with a flattened schema (Appendix S3) and could rate a dependent item without its licensing parent. Structurally valid model output can therefore mismatch the gold set for reasons that are interface artefacts rather than extraction failures.
- **Hierarchical double counting.** Because parent and child levels are counted as separate nodes, a single divergence at the parent level can generate several counted errors.
- **Schema drift.** The gold annotations were produced with a schema prototype that differed slightly from production version 1.0.0, and the benchmark itself used the portable Step-2 schema representation. The values in Table S6 are therefore not directly comparable with the Gemini 3.0 Pro results of the main analysis; they are a differently anchored measurement, not a re-measurement of the same quantity.
- **Small corpus.** Ten datasets from three publications contribute. The estimates are descriptive.

## Sensitivity to model choice and model version

A concern raised in review is that a workflow built on a single proprietary model is inherently sensitive to model version, and that results obtained with one release may not survive the next. The present results speak to this directly. Six models from four providers, spanning frontier and cost-optimized tiers, were run through the identical pipeline without any adaptation of prompts, schema representation or post-processing, and all six produced usable, schema-conform annotations. Five of the six produced F1 values within a band of 5.5 percentage points (61.02% to 66.49%), and only Gemini 3.1 Pro (52.97%) fell clearly outside it.

Two conclusions follow. First, the operating characteristics of this workflow are determined mainly by the orchestration — two-step prompting, landing-page grounding as the source of truth, schema flattening, strict JSON remapping and provenance tracking — rather than by the identity of the underlying model. This is the expected signature of a workflow contribution rather than a modeling contribution, and we now state that framing explicitly in the main manuscript. Second, model substitution is possible without redesign: the pipeline is not tied to a single vendor or a single version, and a model that is deprecated can be replaced by re-running the benchmark reported here, which requires no researcher involvement at all.

Notably, the cost-optimized Flash-tier models were not inferior to the frontier models on this task. Grounded extraction against a controlled vocabulary appears to be constrained more by what the repository landing page actually states than by the reasoning capacity of the model, which has practical consequences for the cost of operating such a pipeline at consortium scale.

## Reproducibility of a non-deterministic pipeline

Repeated execution on identical inputs does not return identical annotations for any of the six models, but the divergence is bounded at roughly 9% to 22% of the annotation set depending on the model. Because several of the underlying sources of non-determinism are outside the caller’s control, we do not claim that pinning temperature and seed would make this pipeline deterministic, and we would caution against reporting such settings as though they constituted a reproducibility guarantee. What can be reported, and what we report here, is a measured consistency band per model, obtained under the same conditions in which the pipeline actually runs.

For practical deployment this suggests reporting the model, the provider, the retrieval date and the measured consistency band together, and treating the annotation output as a versioned artefact with recorded provenance.

## Relation to the main analysis

The supplementary benchmark also provides the head-to-head comparison across models that was absent from the original submission. It does not provide a comparison against dedicated biomedical named-entity recognition tools such as SciSpacy, BioBERT-derived taggers or PubTator. Those tools emit entities from general biomedical vocabularies and do not produce annotations against a hierarchical, CRC-specific controlled vocabulary with conditional item logic, so their output cannot be scored against the present gold standard without an intermediate mapping step that would itself dominate the measured performance. A comparison of that kind is a separate study, and we note it as such rather than reporting a mapping-limited proxy.

# Limitations

1. Dataset detection is evaluated separately and is not incorporated into the entity metrics. All values in Tables S5–S7 are conditional on the dataset having been detected.
2. Entity accuracy is conditional on the 10 gold datasets detected by the models; 2 of the 12 public gold dataset identifiers were not detected.
3. Only three publications contribute to the corpus. Paper-level aggregates are descriptive and do not support inferential modeling at the publication level; for this reason the meta-analytic models reported here are fitted at the dataset level.
4. The entity comparison uses the portable Step-2 benchmark schema and is not directly comparable with the original Gemini 3.0 Pro results reported in the main manuscript.
5. The re-annotation was performed by the research data management team alone. Inter-annotator agreement was not assessed, and no adjudication against the original scientists took place. This is a deliberate feature of the worst-case design, not an oversight, but it means the gold standard reflects one reading of the schema-development annotations.
6. The manually entered custom gold value Macrophage progenitors was excluded because it was not a controlled schema term.
7. The combined manual annotation of the European Nucleotide Archive submission was applied to each of the three public accessions listed in the gold manifest.
8. Exact provider model identifiers were recorded as display names; the routed model version for each call could not be verified from the response payloads at the time of the experiments.

# Data and code availability

The workflow code, including all prompts used in both pipeline steps, is available at <https://github.com/watterm/llm-metadata-annotation/tree/pilot-simplified>. The PILOT metadata schema is published on FreiData (DOI: 10.60493/n9sbn-5ww23).

# References

Engel, F., Giuliani, C., Watter, M., Kalantari, A., Schuller, K., Binder, H., & Kaier, K. (2026a). From manual entry to machine precision: challenges and evolution of metadata schema development in collaborative research centers. *BMC Research Notes*, 19(1), 277. https://doi.org/10.1186/s13104-026-07937-w

Engel, F., Schuller, K., Franzke, C.-W., Giuliani, C., Immler, R., Kalantari Sarcheshmeh, A., Kardoost, A., Kierdorf, K., Proietti, M., Rauer, M., Schulz, C., Watter, M., Binder, H., Börries, M., Marr, C., Wachsmann, A., & Kaier, K. (2026b). *TRR359 metadata schema: A resource for the description of research data in the TRR359 Perinatal Development of Immune Cell Topology (PILOT).* FreiData. https://doi.org/10.60493/N9SBN-5WW23

Kaier, K., Engel, F., Benadi, G., Giuliani, C., Watter, M., Kalantari, A., Schuller, K., Franzke, C.-W., Sperandio, M., & Binder, H. (2026). Identification of biomedical entities from multiple repositories using a specialized metadata schema and search-augmented large language models. *BMC Research Notes*, 19(1), 43.

Miller, J. J. (1978). The inverse of the Freeman–Tukey double arcsine transformation. *The American Statistician*, 32(4), 138. https://doi.org/10.1080/00031305.1978.10479283

Schwarzer, G., & Rücker, G. (2022). Meta-analysis of proportions. In E. Evangelou & A. A. Veroniki (Eds.), *Meta-Research* (Vol. 2345, pp. 159–172). Springer US. https://doi.org/10.1007/978-1-0716-1566-9_10

Thinking Machines Lab. (2025). *Defeating nondeterminism in LLM inference.* https://thinkingmachines.ai/blog/defeating-nondeterminism-in-llm-inference/

Watter, M., Kahle, L., Brunswiek, B., Fichtner, U., Pfaffenlehner, M., Werner, F., Gebele, D., Binder, H., & Knaus, J. (2023). Standardized metadata collection to reinforce collaboration in Collaborative Research Centers. *E-Science-Tage 2023: Empower Your Research — Preserve Your Data.* https://doi.org/10.11588/heidok.00033131
