# Supplementary material for: Accelerating metadata annotation in collaborative research centers: a hybrid AI workflow for biomedical entities
Source: Bioinform Adv. 2026 Sep 23;6(1):vbag283. doi: 10.1093/bioadv/vbag283 (PMC13690486; doi:10.1093/bioadv/vbag283)
Supplement: Supplementary file 1 [file vbag283_supplementary_data.zip › Supplementary Appendix 1.docx]

**Supplementary Appendix 1**

**Detailed Operational Workflow**

The integration of the LLM-augmented annotation into the Research Data Management (RDM) system (*fredato*) follows a multi-stage pipeline consisting of 21 distinct steps. These steps cover data preparation, AI processing, and the human-in-the-loop validation process.

**Phase I: Preparation and Metadata Aggregation**

1. **Load Principal Investigators:** Import PI data (names, emails, project associations).

2. **Load Project Metadata:** Initialize local checkout of the *fredato* metadata repository.

3. **Load Publication List:** Import list of PubMed IDs associated with the Collaborative Research Center.

4. **Retrieve PubMed Metadata:** Query PubMed API for titles, authors, and MeSH keywords.

5. **Convert Metadata:** Transform PubMed responses into *fredato* schema entries.

6. **Add Provenance:** Tag entries with source information.

7. **Filter Entries:** Match imported IDs against existing repository entries to isolate new publications.

8. **Associate Schema:** Schema information is added to new entries.

9. **Commit Changes:** Synchronize metadata baseline to the project repository.

**Phase II: LLM Annotation Prediction**
10. **Dataset Creation:** Associate unique identifiers (UUIDs) with publications.
11. **PDF Acquisition:** Manual download of publication PDFs.
12. **Optical Character Recognition (OCR):** Convert PDFs to Markdown using Mistral OCR or Docling to ensure machine readability.
13. **Execution of Experiments:** Run the two-step LLM prompting strategy (detailed in Section S2).

**Phase III: Integration and Validation**
14. **Output Conversion:** Transform LLM JSON outputs into strict metadata schema entries.
15. **Synchronization:** Commit changes to the *fredato* remote repository.
16. **Notification Management:** Update status sheets for new datasets.
17. **Determine Notifications:** Identify PIs with pending validations.
18. **Email Preparation:** Generate draft emails listing unverified publications.
19. **Staging:** Store drafts for manual quality check.
20. **Distribution:** Send notifications to authors.
21. **Logging:** Update notification timestamps.

**Web Scraping and Data Cleaning Specifications**

To retrieve ground-truth data from repository landing pages, we utilized **Playwright**, a headless browser automation tool. This was necessary to handle modern repositories that rely on client-side JavaScript rendering (DOM manipulation) to display data.

**Retrieval Logic**

1. **Navigation:** The browser navigates to the URL identified by the LLM.

2. **Dynamic Waiting:** The system waits for the DOMContent to load (timeout: 20 seconds) and includes an additional 2-second buffer to allow for late-stage JavaScript updates.

3. **Rate Limiting:** To prevent server overload and blocking, requests are strictly rate-limited to one page visit per second.

**Content Cleaning Rules**

The raw HTML is processed using the LXML library to reduce token usage and noise. The following heuristic cleaning rules were applied:

- **Element Removal:** Headers, navigation bars, footers, scripts, styles, embedded frames, and comments were stripped.
- **Media Filtering:** <img> tags were removed unless they contained an alt attribute (text description).
- **Attribute Filtering:** Only semantic attributes were retained: href, alt, title, name, type, content.
- **Normalization:** Unicode symbols were normalized, and excessive whitespace (newlines, tabs) was collapsed.
- **Truncation:** The final cleaned HTML string was truncated at **30,000 characters** to fit within the LLM context window.

**Fallback Mechanism: URL Reconstruction**

Since the LLM may occasionally generate hallucinated or malformed URLs, a fallback mechanism was implemented. If a scraped URL returns a 404 error, the system validates the Dataset ID extracted by the LLM against known Regular Expression (Regex) patterns.

*Example Fallback Logic (GEO Repository):*

- **LLM Output:** ID: GSE123456, URL: (Broken Link)
- **Regex Check:** Matches pattern ^G(SE|SM|PL|DS)[0-9]+$
- **Action:** Construct correct URL using template: https://www.ncbi.nlm.nih.gov/geo/query/acc.cgi?acc={id}&form=text

**Schema Flattening and Prompt Engineering**

Directly including the full JSON metadata schema in the LLM prompt was avoided to prevent **Context Poisoning** (decreased performance due to excessive token noise) and **Structural Output Complexity** (hallucinations in deep nesting).

**Strategy:**

1. **Tree-like Representation:** The schema is converted into a simplified text tree where every entity is assigned a short ID. The LLM is instructed to use these IDs rather than full field names.

2. **Discriminated Unions:** The output structure is flattened. The LLM specifies the category (e.g., *Organism*, *Intervention*) and provides a flat list of property IDs.

3. **Post-Processing:** A deterministic script maps the LLM's simplified output back into the deeply nested, conditional JSON format required by the *fredato* system.

**Metadata Provenance Strategy**

While the underlying metadata is stored in Git, standard version control is insufficient for granular provenance tracking (e.g., distinguishing between "AI-generated" and "Human-verified" fields within a single commit). To address this, we implemented a JSON-based provenance map within the metadata files.

**Implementation Details:**

- **Object Identity:** Every metadata object is assigned a unique identifier (UUID).
- **Provenance Map:** A top-level provenance object maps these UUIDs to their history. Because simple JSON types (strings/numbers) cannot carry attributes, the map tracks the parent object and its non-object properties.
- **Tracked Fields:**
  - origin: Values include user, ai, or import.
  - model_info: Records the specific model version used for generation.
  - review_status: Tracks the validation lifecycle (pending, accepted, rejected, modified).
- **Implicit Acceptance:** As the user interface allows for streamlined verification, unmodified AI suggestions that are saved by the user are programmatically tagged as accepted.
- **Rejection Handling:** If an object is deleted by the user, the provenance entry is retained but marked as rejected to allow for false-positive analysis.
